# An Improved Adaptive Kalman Filter Positioning Method Based on OTFS

**DOI:** 10.3390/s25196157

**Published:** 2025-10-04

**Authors:** Siqi Xia, Aijun Liu, Xiaohu Liang

**Affiliations:** School of Communication Engineering, Army Engineering University, Nanjing 210007, China; njtzbtt@163.com (S.X.); liangxiaohu688@163.com (X.L.)

**Keywords:** sixth-generation mobile communications systems (6G), complex environments, adaptive Kalman filter, positioning

## Abstract

To mitigate the degradation of positioning accuracy in sixth-generation mobile communication systems under dynamic line-of-sight (LOS) and non-line-of-sight (NLOS) conditions, this paper proposes an improved adaptive Kalman filter positioning method based on Orthogonal Time Frequency Space (OTFS)-modulated signals. Firstly, the distance can be measured by using the OTFS-modulated signals transmitted between base stations and nodes. Secondly, the distance information is converted into the distance difference information to establish the time difference of arrival (TDOA) positioning equation, which is preliminarily solved using the Chan algorithm. Thirdly, residuals are calculated based on the preliminary positioning results, dividing the complex environment into distinct regions and adaptively determining corresponding genetic factors for each region. Finally, the selected genetic parameters are substituted into the Sage–Husa adaptive Kalman filter equations to estimate positioning results. The simulation analysis demonstrates that in complex environments featuring both line-of-sight and non-line-of-sight conditions, the vehicle motion trajectories estimated using this method more closely approximate actual trajectories. Additionally, both the accuracy and stability of positioning results show significant improvement compared to traditional methods.

## 1. Introduction

Location-Based Services (LBSs) have become one of the essential wireless communication applications driving rapid development across various fields [1]. From traditional vehicle route navigation and mobile phone positioning to environmental monitoring, disaster early warning, autonomous driving, and automated parking, all these applications rely on LBSs’ support. In unobstructed outdoor environments, carrier positioning primarily relies on GNSSs (Global Navigation Satellite Systems). However, as future application scenarios grow increasingly complex, indoor and outdoor obstructions and interference become more prevalent, often hindering normal GNSS signal reception. Consequently, relying solely on GNSSs can no longer meet high-precision positioning demands [2], making research into wireless signal-based positioning critically important.

In recent years, researchers have conducted a series of studies on how to utilize wireless signals such as UWB and Bluetooth for positioning. Zhao et al. [3] proposed a signal positioning algorithm based on maximum mutual information entropy and unscented Kalman filtering. He et al. [4] introduced a multi-tag UWB positioning method employing spatiotemporal attention graph neural networks, which significantly enhances the accuracy and robustness of UWB positioning, particularly in NLOS environments. Feng et al. [5] proposed an integrated indoor positioning system combining an inertial measurement unit (IMU) with UWB through Extended Kalman Filter (EKF) and Unscented Kalman Filter (UKF) to enhance robustness and accuracy. Cao et al. [6] proposed a UKF method for IMU-assisted UWB indoor navigation systems. By employing TDOA/IMU fusion positioning, it achieved higher positioning accuracy under both linear and circular motion conditions. Motie et al. [7] proposed a method for TDOA positioning of unmanned aerial vehicles (UAVs) by solving nonlinear equations using the least squares (LS) method. Although the aforementioned methods achieve satisfactory positioning results, they necessitate additional deployment of wireless networks, thereby increasing positioning costs. Furthermore, recent studies have explored using reinforcement learning to assist wireless signal-based position estimation [8,9]. However, such approaches generally require substantial quantities of test training data, leading to high computational complexity for positioning estimation algorithms in complex environments [10].

With the rapid advancement of wireless mobile communication technology, methods for positioning using wireless mobile communication signals have also proliferated. For instance, positioning techniques based on Orthogonal Frequency Division Multiplexing (OFDM) modulation signals are widely employed in fifth-generation mobile communication technology [11,12,13]. However, OFDM suffers from Doppler shift and time-domain spreading effects, which degrade signal transmission quality in highly dynamic environments. Addressing the challenge of wireless mobile communication positioning in complex environments, the Sixth-Generation of Mobile Communications Systems (6G) [14] has introduced the time-delay Doppler communication paradigm. Orthogonal Time Frequency Space (OTFS) modulation [15], an advanced technology aligned with this paradigm, effectively addresses communication challenges in high-dynamic environments, positioning OTFS waveforms as a prime candidate for future 6G communications. Meanwhile, to further enhance positioning capabilities in complex and dynamic environments, researchers have also explored advanced optimization and cooperative strategies. For example, Zhang et al. [16] proposed a Stackelberg game-based multi-agent algorithm for resource allocation and task offloading in MEC-enabled C-ITS, which provides valuable insights into improving system performance in highly dynamic wireless scenarios. These works complement mainstream positioning models such as Time of Arrival (TOA) [17], Angle of Arrival (AOA) [18], and Time Difference of Arrival (TDOA) [19,20]. Among these, the TDOA model calculates precise target locations by accurately measuring signal time differences across multiple receiving nodes [21,22,23]. It effectively distinguishes between direct-path and reflected-path signals, significantly reducing the impact of multipath errors on positioning accuracy [3,24].

In summary, although a variety of wireless signal positioning methods have been developed for complex environments, they still exhibit significant limitations. Some approaches depend on additional network deployment, which inevitably increases costs; others are difficult to adapt to highly dynamic environments; and machine learning-based techniques often require extensive training datasets, leading to high computational complexity. Compared with these existing methods, this paper proposes an improved adaptive Kalman filter positioning method based on 6G-OTFS-modulated signals. The OTFS modulation framework provides robustness against high-mobility and Doppler effects, making it suitable for large-scale dynamic environments, while the improved adaptive Kalman filter enhances resistance to non-line-of-sight (NLOS) interference and improves positioning stability. By combining these two advantages, the proposed method achieves high-precision positioning in complex scenarios, demonstrating clear superiority over traditional approaches. First, distance measurements are performed using OTFS signals transmitted between base stations and nodes. Then, the distance information is substituted into the TDOA equation for Chan solution calculation. Based on the preliminary solution results, the positioning environment is divided into different regions, and corresponding genetic factors are determined. Finally, these genetic factors are incorporated into the adaptive Kalman filter equation to update the positioning results. Simulation results demonstrate that the proposed algorithm achieves significantly improved positioning accuracy and stability compared to traditional methods in complex simulated environments. The main contributions of this paper are as follows:

1. To mitigate the impact of Doppler effects on traditional OFDM ranging in dynamic environments, OTFS modulation is employed for distance estimation. Exploiting its sparse representation in the delay–Doppler domain enables more robust and accurate ranging under high mobility.
2. An improved adaptive Kalman filtering algorithm is proposed, incorporating region partitioning and genetic factor selection. By dynamically adjusting filtering parameters according to different positioning environments, the algorithm achieves enhanced adaptability and robustness, thereby significantly improving localization accuracy in complex scenarios.
3. The simulation results demonstrate that, compared with traditional methods, the method proposed in this article significantly improves positioning accuracy and stability in complex environments, highlighting its strong application potential.

The remainder of this paper is organized as follows. The fundamental theories related to the proposed method are introduced in Section 2. The proposed method is discussed in Section 3. The effectiveness and stability of the proposed algorithm are verified through simulation experiments in Section 4. Finally, in Section 5, the conclusions are presented.

## 2. Fundamental Theory

### 2.1. OTFS Modulation

**Figure 1:**
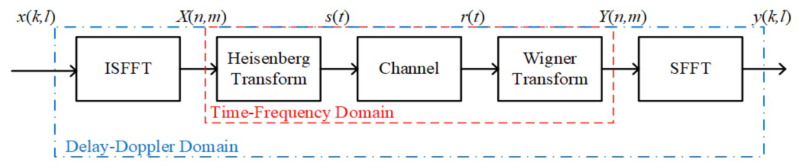
The OTFS signal modulation block diagram.

Suppose that the information to be sent by the delay–Doppler (DD) domain is x[k,l], where k∈[0,M−1] is the sequence number of the time delay domain and l∈[0,N−1] is the sequence number of the Doppler domain. The OTFS signal modulation block diagram is shown in Figure 1. The OTFS sender first uses the ISFFT (Xin finite Fourier inverse transform) to convert the information x(k,l) to be sent from the delay–Doppler domain (DD domain for short) to the time frequency domain (TF domain) signal X(n,m); then it uses the Heisenberg transform to convert the signal X(n,m) in the time–frequency domain into the time-domain signal s(t); after transmission through the channel, it becomes the signal r(t), and at the receiving end uses the Wigner transform to convert the received time domain signal r(t) from the time domain to the TF domain Y(n,m); finally, it uses the SFFT (Xin finite Fourier transform) to convert the information Y(n,m) from the TF-domain to the DD-domain signal y(k,l)

According to the above process, the signal received by the receiving end can be expressed as

(1) $$\begin{array}{c} r\left(t\right)=\int_{\nu }\int_{\tau }h\left(\tau ,\nu \right)x\left(t-\tau \right)e^{j2\pi \nu (t-\tau )}d\tau d\nu +n\left(t\right) \end{array}$$

where h(τ,ν) represents the channel impulse response, which describes the channel response of the channel to pulses with delay τ and Doppler ν, and n(t) is Gaussian white noise.

### 2.2. TDOA Algorithm

Time Difference of Arrival (TDOA) positioning is a method for determining the location of the tag based on the differences in signal arrival times across multiple receivers [19]. Specifically, it estimates the tag position by measuring the relative propagation delays from the transmitter to each base station.

**Figure 2:**
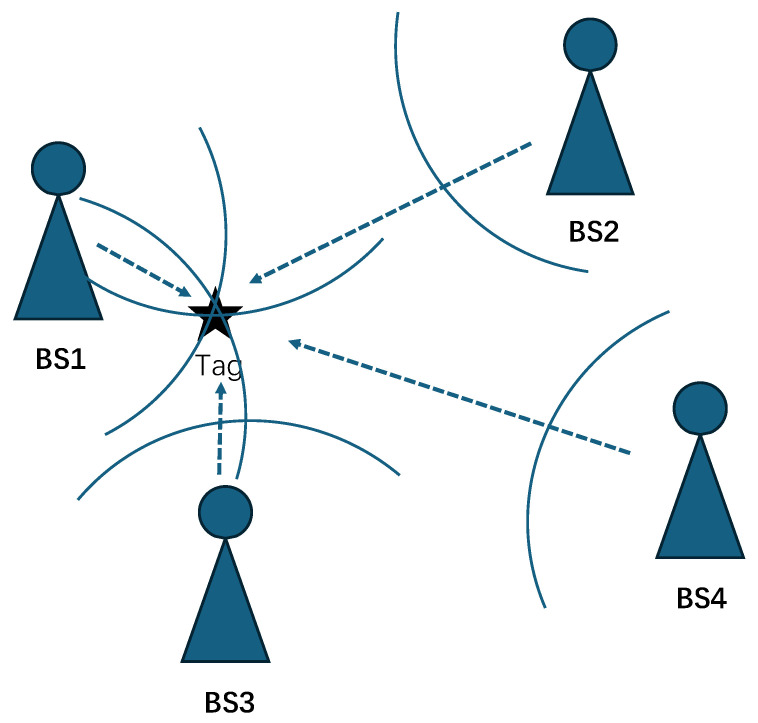
TDOA model.

The TDOA model is shown in Figure 2. Assuming that the coordinate of the tag is (x,y,z) and the coordinate of the *i*th base station (BS) is $\left(x_{i},y_{i},z_{i}\right)$, i=1,2,3,…,n. the following equation can be obtained:

(2) $$\begin{array}{c} D_{i}=\sqrt{{\left(x_{i}-x\right)}^{2}+{\left(y_{i}-y\right)}^{2}+{\left(z_{i}-z\right)}^{2}} \end{array}$$

where $D_{i}$ represents the distance from the tag to the *i*th base station using the OTFS modulation signal.

Based on Equation (2), the following equation can be obtained:

(3) $$\begin{array}{c} D_{i}^{2}-D_{1}^{2}=-2\left(x_{i}-x_{1}\right)x-2\left(y_{i}-y_{1}\right)y-2\left(z_{i}-z_{1}\right)z+K_{i}^{2}-K_{1}^{2} \end{array}$$

where $K_{i}^{2}=x_{i}^{2}+y_{i}^{2}+z_{i}^{2}$, $K_{1}^{2}=x_{1}^{2}+y_{1}^{2}+z_{1}^{2}$.

Let $D_{i1}$ denote the distance difference between the *i*th base station and the tag relative to the first base station. Taking the square of $D_{i1}$, the result is shown in Equation (3):

(4) $$D_{i1}^{2}=D_{i}^{2}+D_{1}^{2}-2D_{i1}=D_{i}^{2}-D_{1}^{2}+2D_{1}^{2}-2D_{i1}$$

Substituting Equation (3) into Equation (4) and performing simplification yield Equation (5):

(5) $$\begin{array}{c} \left(x_{i}-x_{1}\right)x+\left(y_{i}-y_{1}\right)y+\left(z_{i}-z_{1}\right)z\\ =-D_{1}D_{i1}+\frac{1}{2}\left(K_{i}^{2}-K_{1}^{2}-D_{i1}^{2}\right) \end{array}$$

For *i* base stations, Equation (6) can be rewritten in matrix form:

(6) $$\left[\begin{array}{ccc} x_{2}-x_{1} & y_{2}-y_{1} & z_{2}-z_{1}\\ x_{3}-x_{1} & y_{3}-y_{1} & z_{3}-z_{1}\\ \vdots & \vdots & \vdots \\ x_{i}-x_{1} & y_{i}-y_{1} & z_{i}-z_{1} \end{array}\right]\left[\begin{array}{c} x\\ y\\ z \end{array}\right]=-\left[\begin{array}{c} D_{21}\\ D_{31}\\ \vdots \\ D_{i1} \end{array}\right]D_{1}+\frac{1}{2}\left(\begin{array}{c} K_{2}^{2}-K_{1}^{2}-D_{21}^{2}\\ K_{3}^{2}-K_{1}^{2}-D_{31}^{2}\\ \vdots \\ K_{i}^{2}-K_{1}^{2}-D_{i1}^{2} \end{array}\right)$$

There are multiple methods for solving Equation (6), such as the least squares method, Taylor expansion method, and Chan algorithm. Among these, the least squares solution is susceptible to observation noise, resulting in poor coordinate accuracy, while the Taylor expansion method may cause iterative divergence, leading to positioning failure. Therefore, the Chan algorithm is currently the primary method for solving the TDOA positioning model. The Chan algorithm [25] is a two-step weighted least squares method with a closed-form solution. It exhibits excellent performance under Gaussian noise distribution and is widely applied across various scenarios. The main solution process is as follows.

Let $Z_{a}={\left[\begin{array}{cccc} x & y & z & D_{1} \end{array}\right]}^{T}$. Due to the presence of the error vector ε, rewrite Equation (6) as:

(7) $$\begin{array}{c} \varepsilon =h-G_{a}Z_{a} \end{array}$$

where $\;G_{a}=\left(\begin{array}{cccc} x_{2}-x_{1} & y_{2}-y_{1} & z_{2}-z_{1} & D_{21}\\ x_{3}-x_{1} & y_{3}-y_{1} & z_{3}-z_{1} & D_{31}\\ \vdots & \vdots & \vdots & \vdots \\ x_{i}-x_{1} & y_{i}-y_{1} & z_{i}-z_{1} & D_{i1} \end{array}\right),\;h=\frac{1}{2}\left(\begin{array}{c} D_{21}^{2}-K_{2}+K_{1}\\ D_{31}^{2}-K_{3}+K_{1}\\ \vdots \\ D_{i1}^{2}-K_{i}+K_{1} \end{array}\right).$

Assuming the measured noise variance in the TDOA positioning algorithm is $\sigma_{i1}^{2}$, the covariance matrix of the measured noise is

(8) $$\begin{array}{c} Q=\mathrm{diag}\left\{\sigma_{21}^{2},\sigma_{31}^{2},\ldots ,\sigma_{i1}^{2}\right\} \end{array}$$

Equation (7) can be solved by using the weighted least squares method

(9) $$\begin{array}{c} Z_{a}={\left(G_{a}^{T}Q^{-1}G_{a}\right)}^{-1}G_{a}^{T}Q^{-1}h \end{array}$$

In the above process, the covariance matrix *Q* is mainly used instead of the covariance matrix for measuring noise, which increases a certain error value. Therefore, the above process can be further optimized. Reconstructing the error equation system by using the results from Equation (9), $Z_{a}^{\prime }$ can be obtained by performing the weighted least squares estimation again. The related algorithm can be found in [24].

The final estimation is

(10) $$\begin{array}{c} Z_{p}=\pm \sqrt{Z_{a}^{\prime }}+{\left[\begin{array}{ccc} x_{1} & y_{1} & z_{1} \end{array}\right]}^{T} \end{array}$$

where $Z_{p}$ represents the specific position of the tag within the coordinate system.

### 2.3. Adaptive Kalman Filtering

The Adaptive Kalman Filter (AKF) is an extension of the traditional Kalman filter. By dynamically adjusting system model parameters or noise statistical characteristics, it addresses the limitations of conventional methods in time-varying environments (such as model mismatch and noise non-stationarity), thereby enhancing the robustness and accuracy of state estimation [26]. Assume the mathematical model of a linear discrete-time system is

(11) $$\begin{array}{c} x_{k}=Ax_{k-1}+Bu_{k}+Cw_{k} \end{array}$$

(12) $$\begin{array}{c} y_{k}=Hx_{k}+v_{k} \end{array}$$

where *k* is the discrete time, $x_{k}$ denotes the state vector, $y_{k}$ denotes the measurement vector, *A* denotes the state transfer matrix, *C* denotes the noise driver matrix, *H* denotes the measurement matrix, $\omega_{k}$ and $v_{k}$ are uncorrelated zero-mean Gaussian white noise, *B* denotes the control input matrix, and $u_{k}$ denotes the control input vector.

Then, the Sage–Husa Adaptive Kalman Filter (SHAKF) formula is as follows:

(13) $$\begin{array}{cc} \; & {\hat{x}}_{k,k-1}=A{\hat{x}}_{k-1}+Bu_{k-1}+C{\hat{q}}_{k-1}\\ \; & P_{k,k-1}=AP_{k-1}A^{T}+C{\hat{Q}}_{k-1}C^{T}\\ \; & K_{k}=P_{k,k-1}H^{T}{\left[HP_{k,k-1}H^{T}+{\hat{R}}_{k-1}\right]}^{-1}\\ \; & \varepsilon_{k}=y_{k}-H{\hat{x}}_{k,k-1}-{\hat{r}}_{k-1}\\ \; & {\hat{x}}_{k}={\hat{x}}_{k,k-1}+K_{k}\varepsilon_{k}\\ \; & P_{k}=\left[I-K_{k}H\right]P_{k,k-1} \end{array}$$

where $P_{k|k-1}$ and $P_{k|k}$ are the a priori estimation error covariance matrix and the a posteriori state estimation error covariance matrix at moment *k*, respectively, $K_{k}$ is the filtering gain matrix, ${\hat{q}}_{k-1}$ is the mean of the process noise estimation, ${\hat{Q}}_{k-1}$ denotes the covariance of the process noise estimation, ${\hat{r}}_{k-1}$ represents the mean of the measurement noise estimation, ${\hat{R}}_{k-1}$ is the measurement noise covariance matrix estimation, and $R_{0}$ is the nominal measurement noise covariance matrix defined, and $\varepsilon_{k}$ is the measurement noise.

Finally, update ${\hat{q}}_{k},{\hat{Q}}_{k},{\hat{r}}_{k},$ and ${\hat{R}}_{k}$ by calculating the weighting factor

(14) $$\begin{array}{cc} \; & d_{k}=\frac{1-b}{1-b^{k+1}},\;0<b<1\\ \; & {\hat{q}}_{k}=\left(1-d_{k-1}\right){\hat{q}}_{k-1}+d_{k-1}\left({\hat{x}}_{k}-A{\hat{x}}_{k-1}\right)\\ \; & {\hat{Q}}_{k}=\left(1-d_{k-1}\right){\hat{Q}}_{k-1}\\ \; & \;+d_{k-1}\left(K_{k}\varepsilon_{k}\varepsilon_{k}^{T}K_{k}^{T}+P_{k}-AP_{k-1}A^{T}\right)\\ \; & {\hat{r}}_{k}=\left(1-d_{k-1}\right){\hat{r}}_{k-1}+d_{k-1}\left(y_{k}-H{\hat{x}}_{k,k-1}\right)\\ \; & {\hat{R}}_{k}=\left(1-d_{k-1}\right){\hat{R}}_{k-1}+d_{k-1}\left(\varepsilon_{k}\varepsilon_{k}^{T}-HP_{k,k-1}H^{T}\right) \end{array}$$

where *b* is the forgetting factor, and $d_{k}$ is the weighting coefficient.

## 3. Improved Adaptive Kalman Filter Algorithm Based on 6G-OTFS Modulation Signals

In future 6G application scenarios (such as densely built-up areas, tunnels, and low-altitude domains), both LOS and NLOS environments coexist. Particularly in regions with numerous obstacles, carriers experience frequent switching of signal transmission paths when utilizing wireless signals for positioning, resulting in reduced positioning accuracy. To address these challenges, this paper proposes estimating the distance from base stations to nodes under LOS/NLOS conditions using OTFS-modulated signals; This distance is then converted into a distance difference to establish a Time Difference of Arrival (TDOA) positioning model, with preliminary solutions obtained using the Chan algorithm. Subsequently, positioning residuals from NLOS and LOS scenarios serve as the basis for regional segmentation. Appropriate decay factors are selected for each segmented region to enhance the accuracy of positioning estimates from the Sage–Husa adaptive Kalman filter.

### 3.1. OTFS Ranging

When the base station and node in Figure 2 transmit OTFS-modulated signals, the received time-domain signal r(t) is sampled at interval Ts=T∖N to obtain the following signal:

(15) $$\begin{array}{c} b=r\left(tT_{s}\right)=Hs\left(tT_{s}\right)+n\left(tT_{s}\right) \end{array}$$

where $d_{i}$ is a known sequence of information allocated to each subcarrier.

During delay detection, a local signal $c_{t}$ is generated, and according to the following formula, a rough estimate of the delay can be performed.

(16) $$\begin{array}{c} R\left(u\right)=\sum_{t=0}^{N-1}b_{t+u}\cdot c_{t}^{*}\\ \hat{u}=\arg\left\{\max_{u}\left|R\left(u\right)\right|\right\}\\ \tau_{1}=\hat{u}\cdot T \end{array}$$

where $\tau_{1}$ is the time-delay coarse extraction result, and its minimum resolution is the sampling interval *T*.

The above process can only estimate the integer multiple delay. Since the signal delay causes phase changes in the signal, the decimal multiple-delay estimation can be carried out in the frequency domain. After coarse extraction, the received signal $b_{t}$ and the local training sequence $c_{t}$ are converted to the frequency-domain sequence as $B_{i}$ and $C_{i}$. $b_{t}$ and $c_{t}$ differ in the time domain delay τ. After the FFT, the phase difference φ is generated in the frequency domain according to $B_{i}$ and $C_{i}$. The following formula can achieve accurate estimation of time delay:

(17) $$\begin{array}{c} Z_{i}=B_{i}C_{i}^{*}\\ W=\sum_{i=0}^{N-1-\Delta f}Z_{i}\cdot Z_{i+\Delta f}^{*}\\ \hat{\varphi }=\arg\left(W\right)\\ \tau_{2}=\frac{\hat{\varphi }T}{2\pi \Delta f} \end{array}$$

Multiply the obtained time delay by the speed of light to obtain the distance between the base station and the node.

(18) $$\begin{array}{c} D=c(\tau_{1}+\tau_{2}) \end{array}$$

### 3.2. Improved Adaptive Kalman Filter Positioning Method

Measure the distance between *n* base stations and the node using the method described in Section 3.1. Any l(l>4) of them are selected as a group, and there are a total of *Z* combinations with the values as follows [20]:

(19) $$Z=C_{n}^{l}$$

Establish TDOA equations based on each combination; the localization results of these combinations are solved sequentially using Chan’s algorithm, and the resulting residuals are defined as

(20) $$V=\sum_{i=1}^{N}\left[{\hat{D}}_{i}-∥X_{r}-X_{i}∥\right]$$

where $X_{i}$ represents the coordinates of the ith base station, $X_{r}\left(r=1,2,....,Z\right)$ represents the tag coordinates solved for the mth combination of base stations, and ${\hat{D}}_{i}$ represents the distance between the tag node and the ith base station obtained by ranging using OTFS signals.

The larger the residual value obtained in Equation (20), the larger the difference between the distance obtained using OTFS ranging and the distance calculated using the geometric relationship for that region, i.e., the region where the tag is located at that moment has a high probability of being in an NLOS environment. Conversely, the tag is in a region where direct signal transmission is dominant.

By normalizing the residual values calculated at this measurement moment, the environmental factor at the current measurement moment is obtained as

(21) $$\alpha =\frac{min(V_{r})}{max(V_{r})}$$

The environment factor can be used to represent the direct signaling in different regions, which is divided as follows:

(22) $$b=\left\{\begin{array}{c} b_{1},0<\alpha ⩽\alpha_{1}\\ b_{1}+\left(b_{2}-b_{1}\right)\cdot \frac{\alpha_{2}-\alpha_{1}}{\alpha },\alpha_{1}<\alpha ⩽\alpha_{2}\\ b_{2},\alpha_{2}<\alpha <1 \end{array}\right.$$

where $\alpha_{1}$ and $\alpha_{2}$ represent the thresholds for dividing different regions, which can generally be taken as 0.3 and 0.5 [20], $b_{1}$ and $b_{2}$ represent the upper and lower bounds for the genetic factor values, typically set to $b_{1}=0.9,b_{2}=0.98$ [27,28].

**Figure 3:**
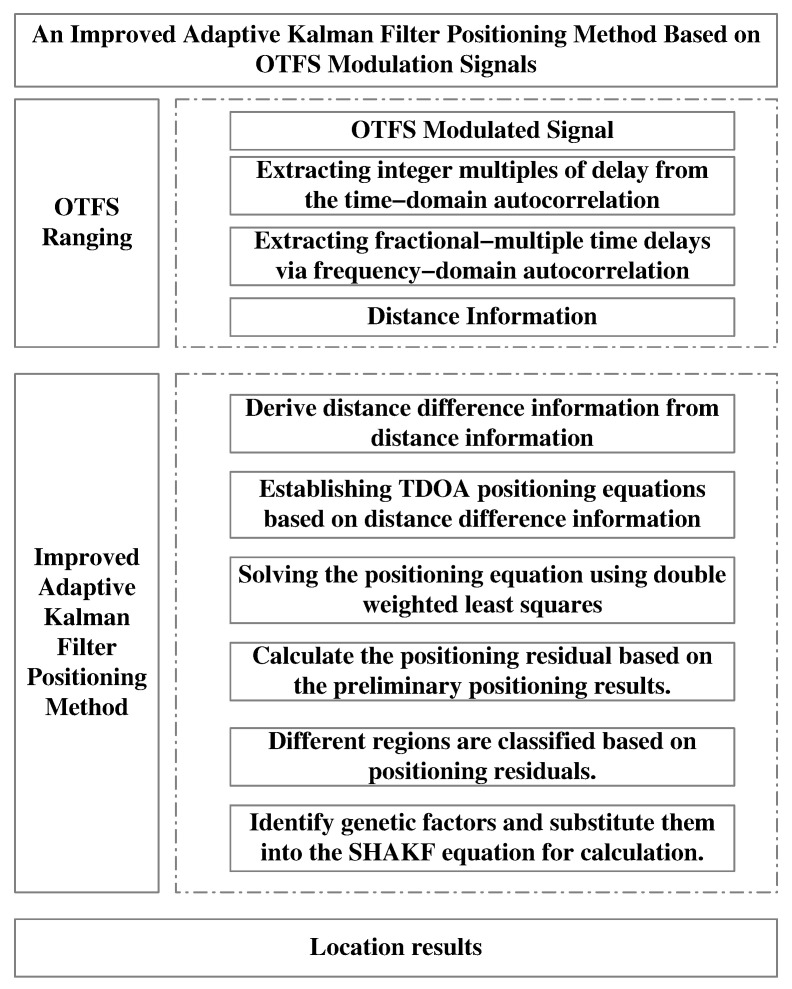
Algorithm flowchart.

Since the value of the genetic factor affects the stability of the filtering results, a smaller genetic factor value increases the dependence of the updated noise covariance matrix $Q_{k}$ on current information, leading to less stable estimation results. Conversely, a larger genetic factor value yields more stable estimation results [29]. As shown in Equation (22), the proposed method adaptively selects genetic factors based on the node’s location within different regions and incorporates the decay factor into the adaptive Kalman filter at the current measurement time to obtain the positioning result at that instant. Therefore, while ensuring positioning accuracy, the proposed method further enhances the stability of positioning results through adaptive selection of genetic factors. The specific method flow is illustrated in Figure 3.

## 4. Simulation Experiments and Analysis

### 4.1. Accuracy Evaluation Metrics

To validate the effectiveness of the proposed method, this paper designed three analyses: OTFS ranging performance analysis, positioning trajectory analysis in three-dimensional space, and positioning accuracy analysis. Before commencing the experiments, precision evaluation metrics for each test were first established. Common positioning accuracy measurement indicators include root-mean-square error (RMSE) and Cramér–Rao Lower Bound (CRLB), calculated as follows [19,30]:

(23) $$\;\mathrm{RMSE}\;=\sqrt{\sum_{i=1}^{n}\frac{1}{n}{\left({\hat{x}}_{i}-x\right)}^{2}}$$

where *x* is the mean value of the coordinate vector, which includes *n* points.

(24) $$\mathrm{CRLB}\approx \frac{T^{2}\cdot c^{2}}{16\pi^{2}\cdot \Delta f^{3}\cdot \mathrm{SNR}}$$

where *c* is the speed of light, Δf denotes the subcarrier spacing, and SNR is the signal-to-noise ratio.

### 4.2. Ranging Performance Analysis

**Figure 4:**
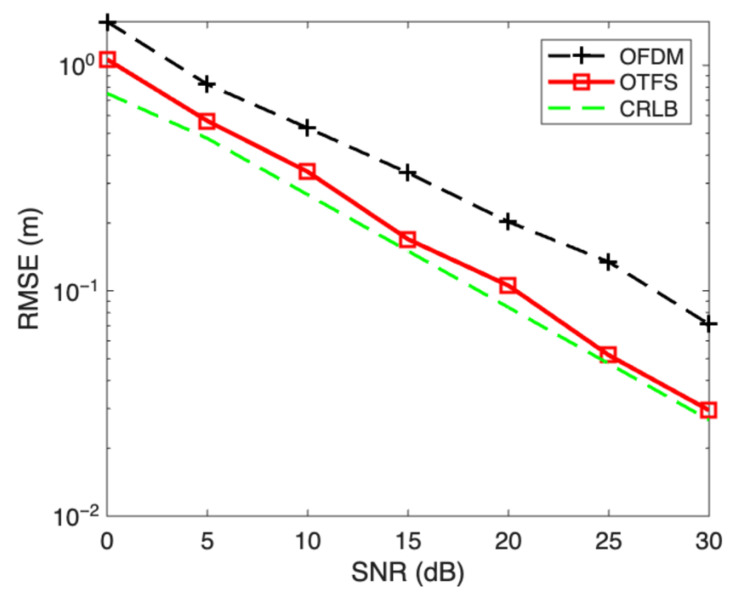
Ranging performance comparison.

Since the method proposed in this paper is based on OTFS-modulated signals—a candidate waveform for future 6G—we first carried out simulation experiments to compare the ranging performance of OTFS-modulated signals with that of OFDM-modulated signals widely applied in 5G. To ensure fairness, the primary simulation parameters were carefully aligned, including a modulation scheme of 4-QAM, subcarrier spacing of 15 kHz, and FFT length of 128 for OFDM. For OTFS signals, the delay–Doppler grid was configured with N = 128 delay points and M = 64 Doppler points, while the OFDM signal adopted a cyclic prefix (CP) length of 32. The multipath channel was modeled with six independent time-domain paths whose maximum delay coordinates were set to 0, 1, 9, 13, 17, and 20, corresponding to delays of 0, 1, 9, 13, 17, and 20 μs, respectively. Each path was also subject to Doppler shifts generated via Jake’s spectrum model to emulate mobility effects. Under these conditions, the ranging performance of both OFDM and OTFS signals was evaluated against the Cramér–Rao Lower Bound (CRLB), with the results presented in Figure 4. This experiment provides the baseline validation of OTFS modulation’s advantages for accurate ranging in highly dynamic multipath environments.

As shown in Figure 4, (1) both the ranging errors of the OFDM-modulated signal and the OTFS-modulated signal decrease as the signal-to-noise ratio increases; (2) the ranging accuracy of OTFS is higher than that of the OFDM-modulated signal and approaches the Cramér lower bound. Therefore, this experiment demonstrates that the OTFS-modulated signal exhibits excellent ranging performance, providing support for subsequent experiments to validate the superior performance of the proposed positioning method.

### 4.3. Performance Analysis of Different Positioning Methods

To further validate the positioning performance of the proposed method in more realistic and complex scenarios, we designed a three-dimensional spatial positioning simulation experiment. The basic simulation parameters are summarized in Table 1. In particular, to explicitly distinguish between line-of-sight (LOS) and non-line-of-sight (NLOS) conditions, the NTN-TDLC and NTN-TDL-B channel models specified in 3GPP TR 38.811zh [30] were adopted, respectively. These standardized models have been widely recognized as representative of urban, suburban, and rural environments, thereby ensuring that the simulation results remain broadly applicable. By deliberately combining four LOS base stations and four NLOS base stations in the spatial layout, we aimed to better reproduce realistic urban-like propagation conditions characterized by frequent LOS/NLOS transitions.

**Figure 5:**
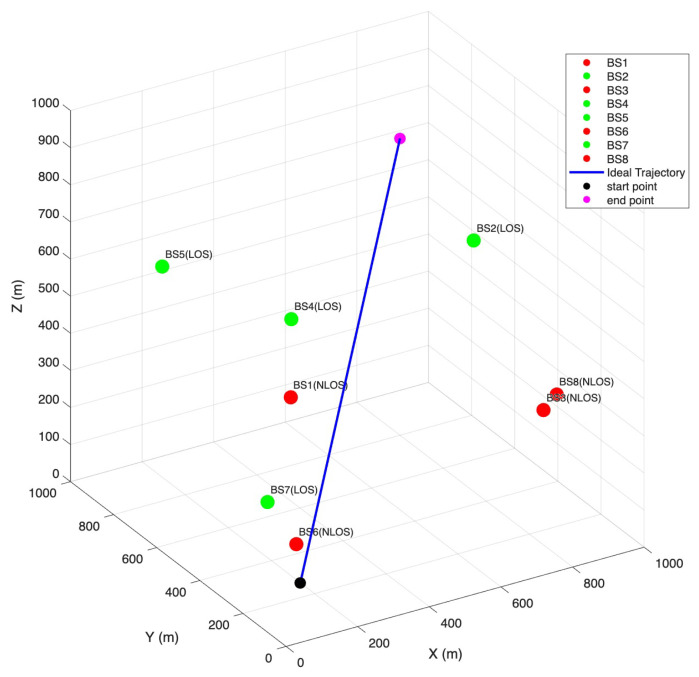
3D Space Base Station Deployment and Node Movement Trajectories.

Figure 5 illustrates the three-dimensional spatial distribution of the eight base stations and their corresponding movement trajectories. To better represent realistic complex environments, four base stations were deliberately placed in line-of-sight (LOS) conditions, while the other four were positioned in non-line-of-sight (NLOS) conditions. This setup ensured a balanced mix of LOS/NLOS paths, allowing the experiment to more faithfully reproduce urban-like communication scenarios characterized by frequent signal blockages and multipath effects. The figure clearly shows how the base stations were uniformly distributed within the simulation space and provides a reference for understanding the subsequent positioning experiments.

**Figure 6:**
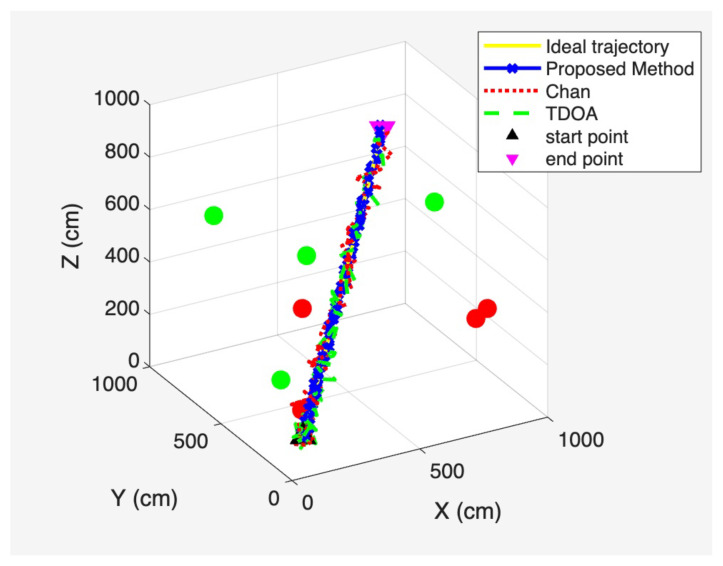
Three-dimensional spatial positioning trajectory.

**Figure 7:**
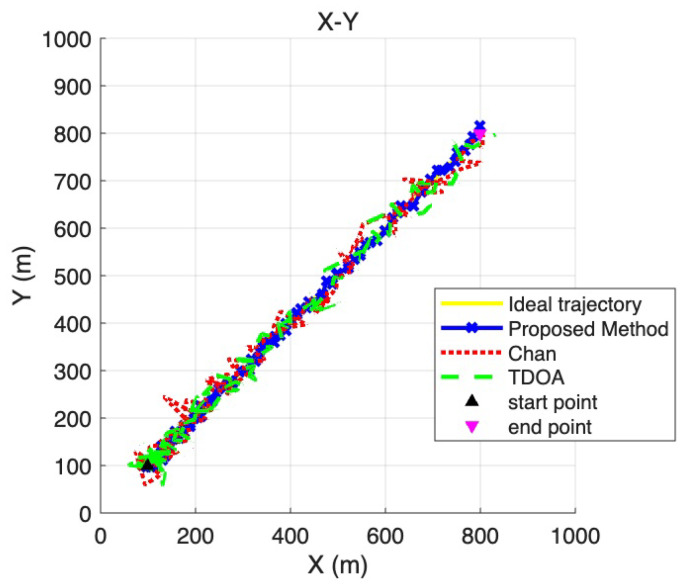
X -Y coordinate plane positioning trajectory.

**Figure 8:**
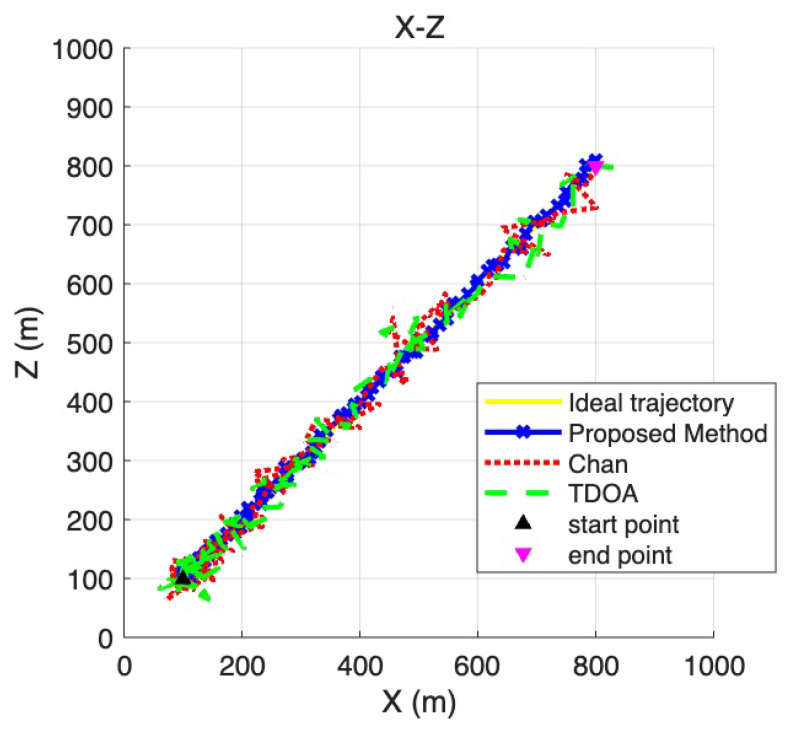
Y -Z coordinate plane positioning trajectory.

**Figure 9:**
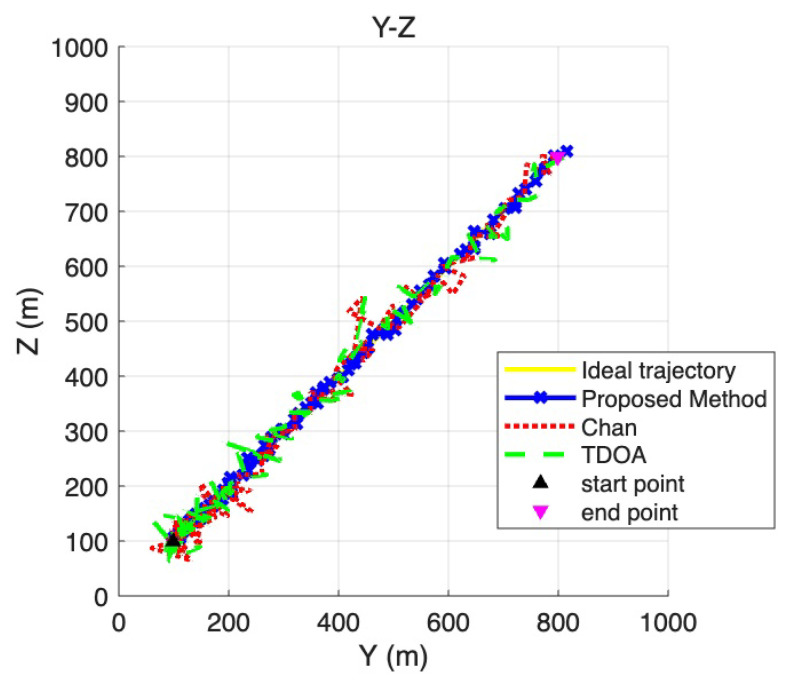
X -Z coordinate plane positioning trajectory.

Based on the spatial distribution of base stations illustrated in Figure 5, Figure 6 presents the overall three-dimensional positioning trajectories obtained using the least-squares TDOA method, the Chan method, and the proposed method under a signal-to-noise ratio (SNR) of 10 dB. This figure provides an integrated view of how different approaches perform when estimating the target trajectory in a mixed LOS/NLOS environment, serving as the basis for the more detailed plane-wise projections shown in Figure 7, Figure 8 and Figure 9.

To provide a more detailed view of the positioning performance across different coordinate dimensions, Figure 7, Figure 8 and Figure 9 present the two-dimensional projections derived from the three-dimensional trajectories shown in Figure 6. Specifically, Figure 7 corresponds to the X–Y plane, Figure 8 to the Y –Z plane, and Figure 9 to the X –Z plane. These projections allow a more fine-grained comparison of the trajectory deviations in each dimension. By examining the fluctuations and offsets across the different coordinate planes, the figures help reveal how the LOS/NLOS environment impacts positioning performance and how the proposed method adapts more effectively to these challenging conditions.

To better compare the positioning accuracy and computational complexity of the three algorithms, Table 2 presents the root-mean-square error (RMSE) and runtime for each positioning algorithm. The RMSE values indicated that the traditional TDOA and Chan algorithms exhibited positioning errors of 2 m and 1.4 m, respectively. The proposed method achieved a positioning accuracy of approximately 0.6 m, surpassing the other two methods by 70% and 57%, respectively, while effectively reducing positioning errors in complex low-altitude environments. In terms of runtime, both traditional TDOA and Chan algorithms completed within 0.1 s. The proposed method, however, involved multiple residual calculations and Kalman filter iterations, increasing computational complexity and extending runtime. Nevertheless, it still fundamentally met real-time positioning requirements. In summary, the proposed method achieved significantly higher positioning accuracy than the other two algorithms and is suitable for real-time positioning environments.

### 4.4. Performance Analysis Under Different Genetic Factors

**Figure 10:**
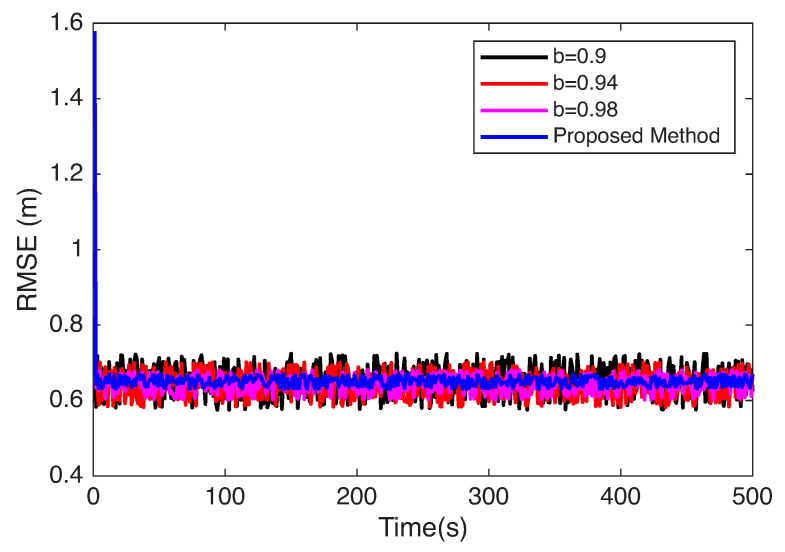
Performance analysis of localization under different genetic factors (parameter b).

The experiments in Section 4.3 were designed to evaluate the localization performance of the proposed method under different conditions. In this setup, a comparative analysis was carried out between the Sage–Husa Adaptive Kalman Filter (SHAKF) localization method configured with different genetic factor values (denoted as parameter b in Figure 10) and the proposed adaptive strategy. The genetic factor b serves as a key parameter that regulates the balance between reliance on current observations and the smoothing effect of historical information in the filtering process. To comprehensively assess its influence, multiple settings of b were considered in the SHAKF method, while the proposed approach adaptively adjusted b across different regions. This experimental configuration was specifically designed to examine how varying genetic factor conditions impact the stability and adaptability of the localization process.

As shown in Figure 10, when a smaller genetic factor is selected, the adaptive Kalman filter estimation results become more dependent on current information. When frequent transitions occur between LOS and NLOS environments, the resulting noise variations cause significant fluctuations in positioning results. Conversely, when a larger genetic factor is employed, the adaptive Kalman filter’s estimation results become less dependent on current information. Consequently, noise variations cause reduced fluctuations in positioning results, but this approach exhibits greater lag in information updates. Since the proposed method adapts the genetic parameter across different regions while leveraging the advantages of varying parameter sizes, it achieves significant improvements in both stability and accuracy, further validating its effectiveness.

## 5. Conclusions

This paper proposed an improved adaptive Kalman filter positioning method based on OTFS-modulated signals. The method consisted of three main parts: first, estimating the distance between the base station and the node using OTFS modulated signals; second, establishing a TDOA positioning model based on the distance information and performing coordinate calculation using the Chan algorithm to divide the positioning area; finally, determining genetic factors according to different areas and then using the Sage–Husa adaptive Kalman filter for node coordinate estimation. Simulation experiments and analysis demonstrated that compared to conventional methods, the proposed approach achieved significantly improved positioning accuracy and stability in environments featuring both line-of-sight and non-line-of-sight conditions. While the method still involves higher computational complexity and has only been validated under simulation conditions, future work will focus on improving efficiency and extending validation to more diverse and realistic environments, thereby further enhancing its practical applicability.

## Figures and Tables

**Table 1:** Simulation parameters.

Simulation Environment	Value
Space size	1000 m × 1000 m × 1000 m
LOS signal and NLOS signal probability	50%
Start point	(100 m, 100 m, 100 m)
End point	(800 m, 800 m, 800 m)
Error distribution	Additive Gaussian white noise
Number of base stations	8

**Table 2:** The RMSE of different algorithms.

Method	RMSE (cm)	Operating Time (s)
TDOA	201.795	0.054
Chan	144.153	0.097
Proposed Method	65.362	0.619

## Data Availability

The original contributions presented in this study are included in this article.
